# Extra-Uterine Pregnancy

**Published:** 1856-06

**Authors:** 


*Extra-Uterine Pregnancy.*

We cut the following from a newspaper. Will Dr. Allaire, or some one of the medical attendants, give us a more complete account of the case?—

"It seems that Mrs. Philip Youngles, who is about thirty years of age, has been suffering from a tumor for several years. While in Germany, some two years after the birth of her first child, she supposed herself to be some seven months in her second pregnancy, when she was seized with what she supposed to be labor pains—which finally subsided, and appearances of pregnancy gradually passed away; leaving, however, a small tumor in her right side. Some two years after, she became pregnant for the third time, and in due season was delivered of a fine, healthy child. She enjoyed her usual good health, removed to this country, and was finally delivered of a third child about a year since. About two months after the birth of the last child, she experienced some pains from the tumor, which became highly inflamed, and finally broke, discharging large quantities of very offensive matter. Last Monday, Dr. Heis, of Du Page county, was called, and Drs. Young and Allaire were called in consultation, when it was determined to open the tumor, from whence they removed the bones of a fœtus of about seven months growth, which were completely hardened."

